# Postoperative pain evaluation in laparoscopic radical prostatectomy surgery using tranexamic acid: analgesia?, hyperalgesia??

**DOI:** 10.1007/s00345-025-05581-w

**Published:** 2025-05-13

**Authors:** Gülten Arslan, Nihan Yaman Mammadov, Ceren Önal, Fırat Mavi, Fatih Doğu Geyik, Banu Eler Çevik

**Affiliations:** 1 Department of Anesthesiology and Reanimation, University of Health Sciences, Kartal Dr Lütfi Kırdar City Hospital, Istanbul, Turkey; 2 Ağrı Training and Research Hospital, Ağrı, Turkey

**Keywords:** Analgesia, Hyperalgesia, Laparoscopic radical prostatectomy, Postoperative pain, Tranexamic acid

## Abstract

**Purpose:**

Tranexamic acid(TXA), an antifibrinolytic agent, is widely used to reduce bleeding, but its effect on pain is not clear.The purpose of this study was to evaluate the effectiveness of intravenous TXA on postoperative pain and bleeding in patients undergoing laparoscopic radical prostatectomy(LRP).

**Methods:**

Our study was conducted as a prospective, observational study. Seventy patients aged 18-75years, ASA II-III, who would undergo LRP surgery under general anesthesia were included in the study.After anaesthesia induction, maintenance was provided with desflurane and 0.1-0.5mcg/kg/min remifentanil infusion with BIS monitoring.The control group(Group C) (*n* = 35) received 100 ml of saline and the study group(Group TXA) (*n* = 35) received TXA 15 mg/kg bolus 10 min before the incision and then 100 mg/hour infusion until skin closure. Demographic, hemodynamic data, ASA, education level, duration of operation and anesthesia, bleeding, administered fluid and remifentanil amounts, hemoglobin values(at the beginning of the operation, 2nd hours, end of the operation, postoperative 12th, 24th hours), Visual Analog Scale (VAS) (at 0th, 6th, 12th, 24th hours postoperatively), time to first rescue analgesia requirement, number of rescue analgesia used within 24h and side effects were recorded.

**Results:**

It was observed that VAS scores were statistically higher in TXA group at postoperative 0th and 6th hours, postoperative rescue analgesia requirement was higher and first rescue analgesia requirement time was shorter. No difference was determined between the groups in terms of other parameters.

**Conclusion:**

In our study, we concluded that there was no significant difference in terms of bleeding in patients who were administered TXA, but this agent may cause hyperalgesia and a special approach to analgesia should be taken in cases where it is decided to be used.

**Clinical Trials.gov:**

(NCT06040853)

## Introduction

Pain and bleeding are the most common perioperative complications. Tranexamic acid (TXA) is an antifibrinolytic agent that prevents fibrin degradation by inhibiting the conversion of plasminogen to plasmin [1]. Recently, TXA, which provides hemostasis in this way, has become widely used in surgical applications to minimize intraoperative blood loss. It is also increasingly used in laparoscopic surgeries where bleeding is important because it affects the visibility of the operation field and provides a great advantage for the surgeon. However, although its effect on bleeding is clearer, its role in postoperative pain management remains unclear. Some studies suggest that TXA may reduce pain by reducing inflammatory responses and tissue compression due to decreased hematoma formation [2]. Conversely, other studies emphasize potential hyperalgesic effects and suggest that TXA’s interaction with neurotransmitter systems such as gamma-aminobutyric acid (GABA) and glycine receptors may increase pain sensitivity [3].

Knowing the effect of TXA in pain modulation is very important to optimize postoperative care, especially in surgeries where pain management is very important. Through a structured observational study, the primary aim of this investigation was to examine whether intravenous TXA administration enhances analgesia or contributes to hyperalgesia in patients undergoing laparoscopic radical prostatectomy (LRP) by assessing variables such as Visual Analogue Scale (VAS) scores, rescue analgesia requirements. Our secondary aim was to investigate the effect on perioperative blood loss.

## Materials and methods

Our prospective, observational study was approved by our Hospital Ethics Committee in line with the principles of Declaration of Helsinki (2023/514/246/18) and registered on Clinical Trials.gov (NCT06040853).

The study included 70 patients aged 18–75 years, BMI ≤ 35 kg/m², American Society of Anesthesiologists (ASA) II-III, who gave written informed consent and underwent LRP under general anesthesia by a surgeon with more than 10 years of LRP experience. Patients with bleeding and coagulation disorders, chronic renal failure, TXA allergy, history of cerebral, coronary and thromboembolic events within 6 months before the operation were excluded.

In patients who underwent standard anesthesia induction after routine noninvasive monitoring, anesthesia was maintained with desflurane with a minimum alveolar concentration value of 1 and remifentanil infusion at 0.1–0.5 mcg/kg/min, targeting 40–60 values with bispecteral index monitoring. The control group (Group C) (*n* = 35) was given 100 ml saline. Based on both the existing literature [4–5] and our clinical experience, 15 mg/kg TXA bolus 10 min before the incision, and 100 mg/h infusion was administered to the study group (Group TXA) (*n* = 35). All patients were given 1 mg/kg tramadol and 1gr paracetamol for analgesia 30 min before the end of the operation. No nerve block or patient-controlled intravenous analgesia (PCIA) was used for postoperative analgesia. When VAS≥4, intravenous dexketoprofen (50 mg) was used as rescue analgesia, and if it was not sufficient, paracetamol (1gr) was administered as a second rescue analgesic.

Demographic and hemodynamic data, ASA, education level, duration of operation and anesthesia, perioperative bleeding, administered fluid and remifentanil amounts, hemoglobin values (at the beginning of the operation, 2nd hours, end of the operation, postoperative 12th, 24th hours), Visual Analog Scale scores (at 0th, 6th, 12th, 24th hours postoperatively) (VAS assessment was performed by a blinded health worker), time to first rescue analgesia requirement, number of rescue analgesia used within 24 h and side effects were recorded.

### Statistical analysis

Data were entered into the Statistical Package for the Social Sciences (IBM^®^ SPSS Statistics for Windows, Version 23.0, Armonk, NY, USA) software package. Descriptive statistics were used and quantitative variables were characterized using mean, maximum, and minimum values, and percentages were used for qualitative variables. It was decided whether the distributions were normal or not by Kolmogorov-Smirnov analysis.

Normal distributions were reported as mean values, standard deviations (SD) were calculated, and Student’s t-test was used for comparisons between groups. Nonparametric continuous variables were recorded as medians and compared using Mann-Whitney U tests. In the values ​​recorded as median, the Inter Quantile Range (IQR) result was also given. Pearson’s chi-square test was used for comparative analysis of qualitative variables; However, if the sample size was small (≤ 5), Fisher’s exact test was used. Wilcoxon signed-rank test was used to test the significance of the difference between the values ​​of two related measurement groups within the groups and to compare differences in pain measures (VAS scores) between two time points within the same group. *p* value < 0.05 was considered statistically significant. In comparing the paired measurements within the same group, the number of samples required for 85% test power was found to be 35 using the GPower 3.1 package program.

## Results

Demographic, characteristic, clinical features of the patients and their comparison between the groups are given in Table 1. No statistically significant difference was found between the groups in terms of these parameters (*p* > 0.05). No significant difference was determined between the groups in perioperative hemodynamic data, administered fluid, and remifentanil amounts, and side effects.

**Table 1:** Demographic, characteristic and clinical features of the patients

Variables	Total (*n* = 70)	Group C (*n* = 35)	Group TXA (*n* = 35)	*P* value
**Age**,** years**,** mean ± SD**	64,3 ± 6,0	64,8 ± 5,6	63,8 ± 6,4	0,503
**BMI**,** kg/m²**,** mean ± SD**	26,2 ± 2,6	25,9 ± 2,7	26,5 ± 2,3	0,501
**ASA status**,** n/%** **I** **II** **III**	4 / 5,7 47 / 67,1 19 / 27,1	2 / 5,7 22 / 62,9 11 / 31,4	2 / 5,7 25 / 71,4 8 / 22,9	0,717
**Education level**,** n/%** **Primary/Middle School** **High School/University**	39 / 55,7 31 / 44,3	19 / 54,3 16 / 45,7	20 / 57,1 15 / 42,9	0,810
**Duration of anaesthesia**, **min**,** mean ± SD**	247,0 ± 45,8	239,1 ± 45,9	254,8 ± 45,0	0,153
**Duration of surgery**, **min**,** median/IQR**	187 / 65	180 / 75	210 / 60	0,273

***Group C =*** *Control group*, ***Group TXA =*** *Tranexamic acid group*, ***BMI;****body mass index*, ***n;****number*, ***m;****meters*, ***kg***; *kilogram*, ***ASA;****American Society of Anesthesiologists*, ***min;****minute*, ***SD;****standard deviation*, ***IQR;****interquartile range*

When VAS values were compared, it was found that VAS levels at 0th and 6th hours were statistically lower in the C group than in the TXA group (*p* < 0.001 and *p* = 0.004, respectively). However, there was no statistical difference between the two groups in terms of 12th and 24th hour VAS levels (*p* = 0.230 and *p* = 0.146, respectively) (Table 2).

**Table 2:** Comparison of postoperative visual analogue scale between groups

Postoperative VAS	Group C (*n* = 35)		Group TXA (*n* = 35)			*p* value
	Median /IQR	Mean / SD	Median /IQR	Mean / SD		
**0th hour**	2/2	2,25 / 1,82	5/5	4,65 / 2,94		**< 0**,**001**
**6th hour**	2/1	2,62 / 1,37	4/3	3,94 / 2,08		**0**,**004**
**12th hour**	2/1	2,40 / 1,39	2/2	97 / 1,87		0,230
**24th hour**	2/2	1,88 / 0,99	2/1	2,34 / 1,08		0,146
**Comparison within**						
**Group C**					**p value**	
0th hour versus 6th hour					0,156	
6th hour versus 12th hour					0,378	
12th hour versus 24th hour					**0**,**01**	
**Comparison within**						
**Group TXA**					**p value**	
0th hour versus 6th hour					0,164	
6th hour versus 12th hour					**0**,**004**	
12th hour versus 24th hour					**0**,**006**	

***Group C*** *= Control group*, ***Group TXA*** *= Tranexamic acid group*, ***n;****number*, ***VAS;****Visual Analogue Scale*, ***IQR;****interquartile range*, ***SD***; *standart deviation*

When VAS values were compared within groups, there was no statistical difference between 0th hour 6th hour and between 6th hour and 12th hour in C group. But the 12th hour VAS value was statistically higher than the 24th hour VAS value (*p* = 0.01).

When VAS values were compared within the group in the TXA group, no statistical difference was observed between hours 0th and 6th, whereas the VAS value at 6th hours was higher than the VAS value at 12th hours and the VAS value at 12th hours was higher than the VAS value at 24th hours (*p* = 0.004 and *p* = 0.006, respectively).

The time to first rescue analgesia requirement, the number and rate of patients rescue analgesia requirement, and the number of rescue analgesics within the first 24 postoperative hours are shown in Table 3. As seen in Table 3, the number of patients rescue analgesia requirement (*p* < 0.001), the number of rescue analgesics within the first 24 h postoperatively (*p* = 0.005) were higher and the time to first rescue analgesia requirement was shorter (*p* = 0.001) in the TXA group.

**Table 3:** Comparison of the groups in terms of postoperative analgesia requirement

Variables	Group C (*n* = 35)	Group TXA (*n* = 35)	*P* value
**Rescue analgesia requirement**,** n/%**	18 / 51,4	32 / 91,4	**< 0**,**001**
**Time to first rescue analgesia requirement**,** min**,** median/IQR**	210 / 336	37,5 / 75	**0**,**001**
**Number of rescue analgesics within 24 h**,** median/IQR**	1 / 1	2 / 2	**0**,**005**

***Group C =*** *Control group*, ***Group TXA*** *= Tranexamic acid group*, ***n;****number*, ***IQR;****interquartile range*

When the hemogram values were analyzed at the beginning of the operation, at the 2nd hour, at the end of the operation, at the postoperative 12th hour and 24th hour, these values were 12.8±1.3 gm/dL, 12.4±1.2 gm/dL, 11.9±1.0 gm/dL, 11.7±1.0 gm/dL, 12.0±1.2 gm/dL for group C and 13.0±0.7 gm/dL, 12.5±0.8 gm/dL, 12.2±1.1 gm/dL, 12.0±1.0 gm/dL, 12.4±1.0 gm/dL for group TXA, respectively. There was no statistical difference between the groups in terms of hemogram values and the amount of blood transfusion and bleeding at any time point (*p* > 0.05). The blood transfusion rate was 5.7% (*n* = 2) in the C group and 2.9% (*n* = 1) in the TXA group (*p* = 1,000). While the average amount of bleeding in the C group was 348 ± 167 ml, it was 342 ± 260 ml in the TXA group (*p* = 0.922).

## Discussion

Pain management is a factor that cannot be ignored in ensuring rapid recovery of patients after surgery. Our study provides a perspective on the effects of TXA on postoperative pain management in LRP patients. The analysis indicates that while TXA maintains perioperative hemostasis, its impact on pain requires cautious interpretation. The fact that the VAS scores recorded at the 0th and 6th postoperative hours and the need for additional analgesics within 24 h were significantly higher in the TXA group than in the control group indicate potential hyperalgesic effects. In addition, the number of patients requiring additional analgesics was higher in the TXA group than in the control group, and the median time to the first analgesic requirement was shorter. This trend again highlights the increased pain response in the TXA group and requires further investigation of its analgesic.

While we acknowledge that TXA has been widely studied in different surgical fields, especially orthopaedics and cardiovascular surgery, our literature review and analysis focused specifically on the minimally invasive nature of LRP, which presents different physiological and haemodynamic challenges compared to open or overly invasive procedures. When reviewing the available literatures, we observed that the effects of TXA used perioperatively on pain are still unclear. Some studies have shown that TXA can reduce pain and bleeding when used intravenously or intraarticularly in commonly used arthroscopic surgeries [2, 6–11]. They attributed this to TXA’s ability to reduce local compression that causes pain by reducing haematoma and intra-articular effusion and to suppress inflammatory markers such as CRP and interleukin 6, which are necessary for blood resorption.

Reports of beneficial effects of TXA in orthopaedic and cardiovascular surgeries do not mean that the same effect will be observed in LRP. Because perioperative blood loss is relatively low during LRP, the systemic effects of TXA may become more pronounced. We also think that pain mechanisms in LRP may be caused by factors such as pneumoperitoneum and visceral manipulation rather than large tissue trauma.

On the other hand, Remerand et al. [12] reported that although TXA reduced haematoma volume by 30%, it did not affect postoperative pain and may even cause hyperalgesia due to increased morphine consumption on postoperative day 7. Some researchers have also found that TXA administered intravenously or locally in arthroscopic and laparoscopic sleeve gastrectomy operations did not affect perioperative outcomes, including bleeding and postoperative pain [13–16].

Since the tissues surrounding the prostate contain large venous sinuses and have rich blood supply, the pain results of our study, in which we applied TXA to reduce bleeding and clarify the surgical field of view, were found to be compatible with the results of the researchers [17–21]. Ohashi et al. reported that TXA administered intrathecally or intraperitoneally in rats produced behaviours indicative of spontaneous pain and mechanical allodynia and did so by inhibiting GABAA and glycine receptors in the posterior horn of the spine [3]. Barret et al. also found that TXA may cause hyperalgesia by increasing urokinase-mediated plasmin production, possibly in a fibrin-independent manner, and may lead to significant inflammatory C5a elevations at the wound site [18].

Since the occurrence and perception of pain is very complex, the results of these studies may vary. At the same time, pain is a symptom associated with many factors. Therefore, in our study, we tried to provide robust comparative data by applying the same anaesthesia protocols to all groups and standardising as much as possible other variables (age, ASA, education level, etc.) that may affect pain or haemodynamic responses. The potential hyperalgesic effect of TXA may be due to its effect on GABA and glycine receptors, since these neurotransmitters are involved in nociceptive regulation. Blocking these receptors may enhance pain perception by inhibiting inhibitory pathways in the central nervous system. It is thought that this mechanism may become particularly pronounced in surgery of organs with high blood supply, i.e. interventions such as LRP.

Is our study, haemodynamic monitoring and perioperative parameters indicating the amount of bleeding revealed that bleeding was minimally lower in the TXA group, but the difference was not statistically significant. These results support the idea that TXA controls blood loss to some extent, as reported by some investigators [13–14].

To better tailor pain management strategies, future studies could investigate the dose-dependent effects of this drug or potential hyperalgesia-neutralizing adjunctive treatments. Additionally, studies comparing surgical techniques in the same type of surgery to see how the effects of TXA on pain may vary across patient populations will also help improve clinical protocols. Although the efficacy of TXA in reducing surgical blood loss is mostly documented, the fact that it causes hyperalgesia, as demonstrated by higher VAS scores and increased analgesic requirements, may pose a challenge in its use. This study emphasises the importance of considering this feature of TXA in the clinical use of the drug. In conclusion, it was concluded that individualised patient assessment and postoperative care including additional analgesic measures to reduce the hyperalgesic effects of this agent when TXA is administered may be beneficial, especially in surgeries where pain management is important.

### Limitations

Our study was conducted in a limited number of patients and single center. Further multicenter studies with larger sample groups will increase the generalizability of the data obtained. One of the limitations of our study is the potential for confounding due to lack of randomisation. Our study does not determine the optimal dose of the drug. When we look at the literature, it is seen that different doses and routes of administration of TXA are also used. In addition, haemogram and VAS follow-up may be performed for a longer period.

## Data Availability

No datasets were generated or analysed during the current study.
